# The dresden platform is a research hub for ultra-high dose rate radiobiology

**DOI:** 10.1038/s41598-023-46873-8

**Published:** 2023-11-23

**Authors:** Josefine Metzkes-Ng, Florian-Emanuel Brack, Florian Kroll, Constantin Bernert, Stefan Bock, Elisabeth Bodenstein, Michael Brand, Thomas E. Cowan, René Gebhardt, Stefan Hans, Uwe Helbig, Felix Horst, Jeannette Jansen, Stephan D. Kraft, Mechthild Krause, Elisabeth Leßmann, Steffen Löck, Jörg Pawelke, Thomas Püschel, Marvin Reimold, Martin Rehwald, Christian Richter, Hans-Peter Schlenvoigt, Ulrich Schramm, Michael Schürer, Joao Seco, Emília Rita Szabó, Marvin E. P. Umlandt, Karl Zeil, Tim Ziegler, Elke Beyreuther

**Affiliations:** 1 Helmholtz-Zentrum Dresden–Rossendorf, Dresden, Germany; 2 TUD Dresden University of Technology, Dresden, Germany; 3 OncoRay - National Center for Radiation Research in Oncology, Faculty of Medicine and University Hospital Carl Gustav Carus, TUD Dresden University of Technology, Helmholtz-Zentrum Dresden–Rossendorf, Dresden, Germany; 4 Center for Regenerative Therapies (CRTD), TUD Dresden University of Technology, Dresden, Germany; 5 Cluster of Excellence - Physics of Life, TUD Dresden University of Technology, Dresden, Germany; 6 German Cancer Research Center (DKFZ), Heidelberg, Germany; 7 Present Address: Centre Hospitalier Universitaire Vaudois (CHUV), Lausanne, Switzerland; 8 Department of Radiotherapy and Radiation Oncology, Faculty of Medicine and University Hospital Carl Gustav Carus, TUD Dresden University of Technology, Dresden, Germany; 9 German Cancer Consortium (DKTK), partner site Dresden, and German Cancer Research Center (DKFZ), Heidelberg, Dresden, Germany; 10 National Center for Tumor Diseases (NCT/UCC), Dresden, Germany: German Cancer Research Center (DKFZ), Heidelberg, Germany; Medizinische Fakultät and University Hospital Carl Gustav Carus, TUD Dresden University of Technology, Dresden, Germany; Helmholtz-Zentrum Dresden-Rossendorf, Dresden, Germany; 11 Faculty of Physics and Astronomy, Ruprecht-Karls-University, Heidelberg, Germany; 12 ELI ALPS, ELI-HU Non-Profit Ltd., Szeged, Hungary; 13 Department of Oncotherapy, University of Szeged, Szeged, Hungary

**Keywords:** Plasma-based accelerators, Translational research, Preclinical research, Radiotherapy

## Abstract

The recently observed FLASH effect describes the observation of normal tissue protection by ultra-high dose rates (UHDR), or dose delivery in a fraction of a second, at similar tumor-killing efficacy of conventional dose delivery and promises great benefits for radiotherapy patients. Dedicated studies are now necessary to define a robust set of dose application parameters for FLASH radiotherapy and to identify underlying mechanisms. These studies require particle accelerators with variable temporal dose application characteristics for numerous radiation qualities, equipped for preclinical radiobiological research. Here we present the dresden platform, a research hub for ultra-high dose rate radiobiology. By uniting clinical and research accelerators with radiobiology infrastructure and know-how, the dresden platform offers a unique environment for studying the FLASH effect. We introduce its experimental capabilities and demonstrate the platform’s suitability for systematic investigation of FLASH by presenting results from a concerted *in vivo* radiobiology study with zebrafish embryos. The comparative pre-clinical study was conducted across one electron and two proton accelerator facilities, including an advanced laser-driven proton source applied for FLASH-relevant *in vivo* irradiations for the first time. The data show a protective effect of UHDR irradiation up to $10^{5}\text{Gy}/\text{s}$ and suggests consistency of the protective effect even at escalated dose rates of $10^9\text{Gy}/\text{s}$. With the first clinical FLASH studies underway, research facilities like the dresden platform, addressing the open questions surrounding FLASH, are essential to accelerate FLASH’s translation into clinical practice.

## Introduction

The recent observation of a normal tissue protecting effect of ultra-high dose rate (UHDR, mean dose rate $>40{Gy}/{s}$, rapid dose delivery times of $\le 500$ ms)^1,2^ radiation at unchanged tumor treatment efficacy, the so-called FLASH effect^3,4^, promises great benefits for radiotherapy (RT) patients through reduced side effects and increased quality of life after treatment. Since the first description of the FLASH effect^3^, preclinical studies have confirmed the effect for electrons, photons, protons, and carbon ions in various tumor and normal tissue models (reviewed in the literature^1,2,5^). Clinical trials in animals and humans are underway^6^. Yet, two fundamental questions remain to be answered: Firstly, what are the mechanisms causing the FLASH effect, and secondly, what are the dose application parameters required for triggering FLASH? A wide range of mechanisms potentially responsible for FLASH are currently under investigation, including increased radioresistance through oxygen depletion^7,8^, radiochemical reactions^2,9,10^, and immunological as well as cellular effects^11,12^.

The time scale on which the dose is applied to the tissue is discussed as one of the most relevant dose application parameters to trigger the FLASH effect. In this context, two definitions of dose rate are commonly used that together characterize the temporal dose delivery. Both are determined by the beam parameters of the used accelerator: The peak dose rate is the dose rate of a single (particle) bunch, whereas the mean dose rate is the ratio of the total dose to the total dose application time. Moreover, the total dose, dose fractionation, and the applied radiation type can play a role. A survey of FLASH studies, comprising experiments where the FLASH effect was successfully triggered as well as studies where FLASH was not observed, hypothesized one potential set of optimal dose application parameters for FLASH RT^13^. But the influence of the various dose application parameters—from peak and mean dose rate to fraction size and number—is the subject of current research^14–16^.

To determine a robust set of dose application parameters for clinical use, e.g., in FLASH RT, systematic studies are required. These studies ask for particle accelerators that provide electrons, photons, protons, and potentially other ion species, offering a broad range of mean and peak dose rates via widely tunable beam parameters^14,17^. The mean dose rates should ideally cover the entire range from clinically established continuous beam delivery of several $\text{Gy}/\text{min}$ to UHDR, with the option of achieving the same mean dose rate at different peak dose rates and hence temporal beam structures. In this way, the entire cascade of physical, chemical, and biological reactions that follow the incidence of ionizing radiation on a biological sample can be studied^7^.

Research accelerators, as opposed to specialized clinical machines, offer the tunability required for FLASH studies^1^, but additionally need to provide the research environment supporting radiobiological experiments. This entails beam transport and radiation field formation to provide pre-defined dose distributions at an in-air irradiation site, beam monitoring, as well as dosimetry and infrastructure to handle biological samples.

**Figure 1:**
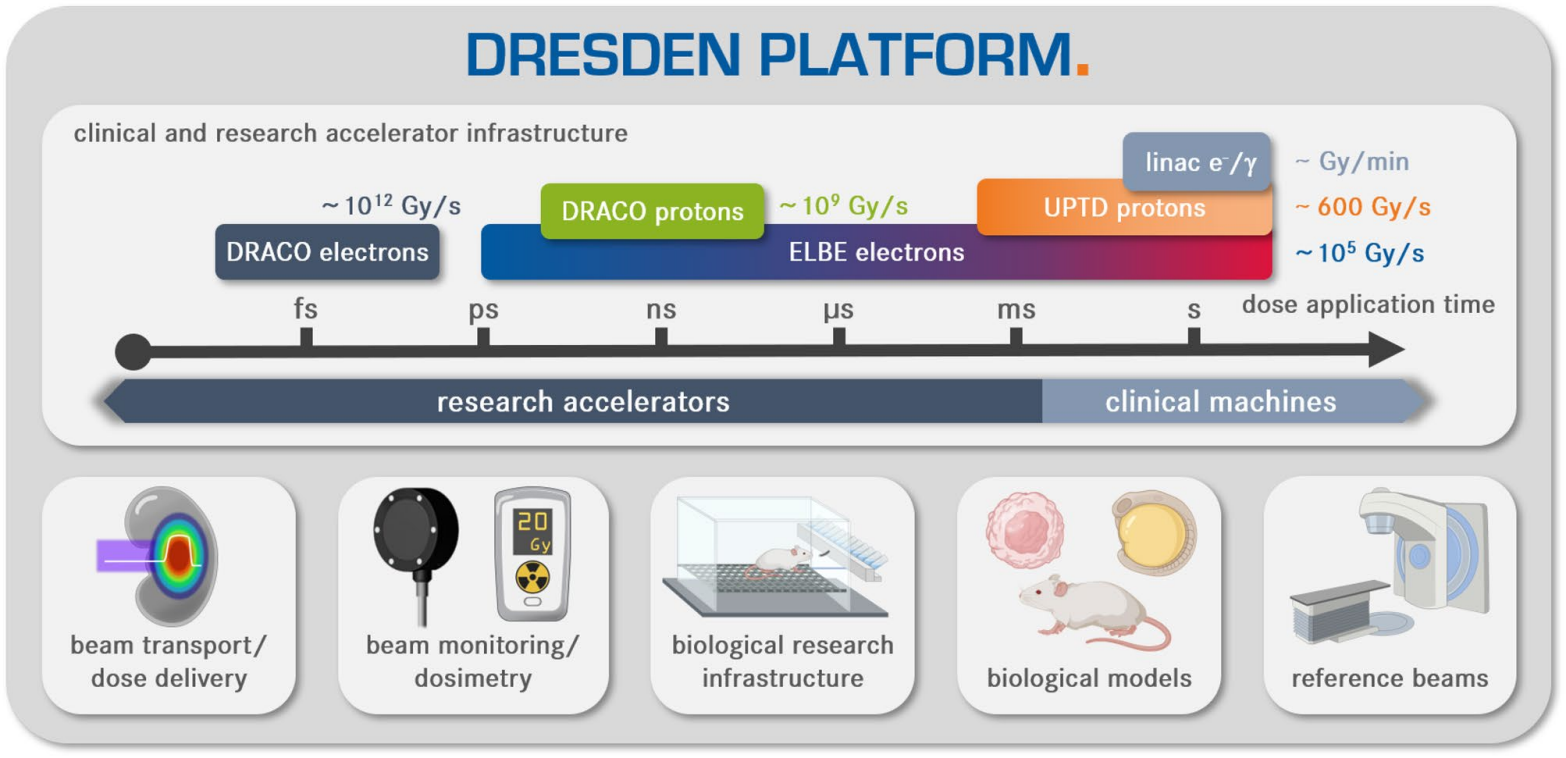
Graphical representation of the dresden platform, a research hub for ultra-high dose rate radiobiology. At the top stands the available clinical (linacs, UPTD) and research accelerator (DRACO, ELBE) infrastructure (see section 2 for facility details). The accelerators are sorted by particle type and dose application time, which ranges from femtoseconds to minutes. For each accelerator, the maximum achievable mean dose rate is noted. Each facility is equipped for radiobiological experiments, providing the necessary technology and know-how from beam transport and dose delivery over beam monitoring and dosimetry to radiobiology infrastructure. The diversity in available dose application parameters is flanked by comprehensive biological models and reference beams to enable comparative radiobiological studies on UHDR effects such as FLASH across the dresden platform. Pictograms created with BioRender.com.

The dresden platform (Fig. 1) as a research hub for UHDR radiobiology fulfills these requirements and offers a unique environment for radiobiological studies in the FLASH regime. The dresden platform extends across the institutions of the Department of Radiotherapy and Radiation Oncology at the University Hospital Carl Gustav Carus, the OncoRay - National Center for Radiation Research in Oncology and the Helmholtz-Zentrum Dresden–Rossendorf to merge clinical and research accelerators providing electron and proton beams with broad-ranging dose application parameters. Clinical machines offer an established infrastructure with standardized irradiation protocols, dosimetry methods, and quality assurance, which favors them as reference sources for studies at research accelerators and for establishing new radiobiological models^18,19^. The dresden platform comprises clinical electron linear accelerators (linacs) and the proton cyclotron of the University Proton Therapy Dresden (UPTD). From this cyclotron, proton beams beyond the clinical setting, i.e., mean dose rates up to $\sim 600\text{Gy}/\text{s}$, are available in a dedicated experimental area^20^. The research accelerators extend the mean dose rate spectrum to $\sim 10^{5}\text{Gy}/\text{s}$ for electrons provided by the ELBE accelerator and $\sim 10^{9}\text{Gy}/\text{s}$ for protons provided by advanced accelerator technology based on laser-plasma acceleration at the DRACO laser^21^. The platform further hosts an experimental facility for laser-plasma acceleration of electron beams of high quality^22,23^ that sets the upper limit of the mean dose rate to $10^{12}\text{Gy}/\text{s}$. Apart from the latter, all accelerators of the dresden platform have been qualified and applied for radiobiological *in vivo* studies^24–26^.

In this paper, we introduce the experimental capabilities of the dresden platform by presenting results from a concerted *in vivo* radiobiology study applying ELBE electrons as well as UPTD and DRACO protons in UHDR and reference irradiation regimes. The study uses zebrafish embryos (ZFEs) as a small animal vertebrate model for acute normal tissue reactions^4,18,27,28^. The specific handling requirements of ZFEs, i.e., aqueous environment, temperature, and timing, were addressed by appropriate setups and suitable workflows at all experimental sites. Site-specific solutions for beam delivery, radiation field formation, beam monitoring, and dosimetry, fulfilling the requirements set by the ZFE model, enable the comparison of radiobiological data acquired across the platform. Moreover, measurements of partial oxygen levels before and during irradiation were integrated into the respective setups for monitoring purposes and to investigate potential oxygen-dependent mechanisms of the FLASH effect^8,29^. For DRACO protons, the study serves as commissioning for FLASH-relevant irradiations of normal tissue models (here ZFE) with synchronous oxygen depletion analysis. Overall, the presented radiobiological data qualifies the dresden platform for further systematic radiobiological studies investigating UHDR radiation in the context of the FLASH effect with unique variable irradiation regimes up to unprecedented dose rates of up to $10^{9}\text{Gy}/\text{s}$.

## The dresden platform

### Accelerators

**Figure 2:**
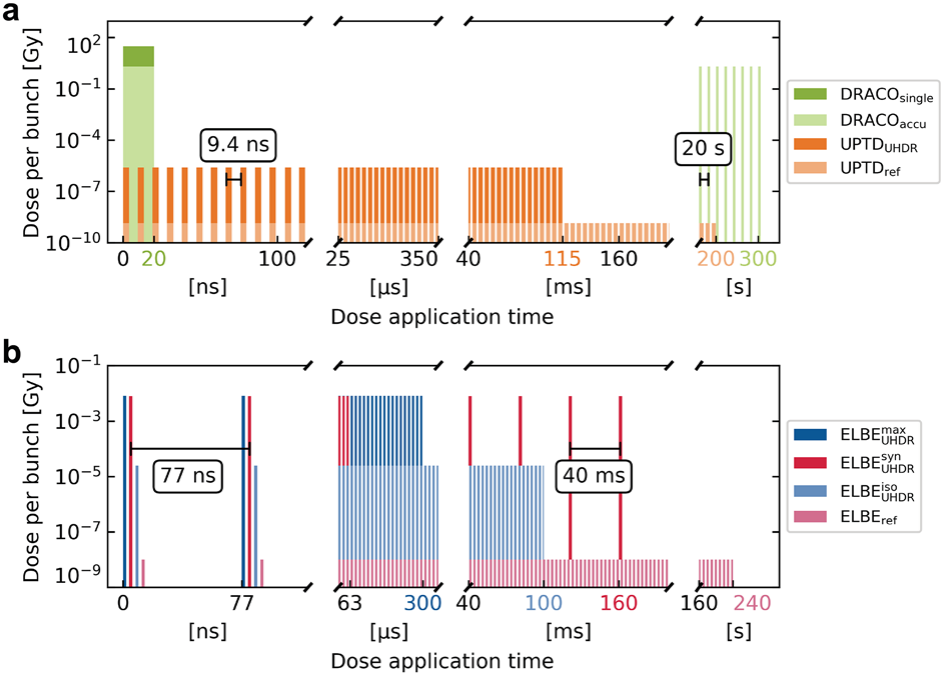
Graphical representation of the temporal sequence of the beam bunches of different irradiation regimes across the dresden platform accelerator landscape. Colored *x*-axis labels mark the dose application time for the delivery of $\sim 30\text{Gy}$ prescribed dose to a ZFE sample in the correspondingly colored irradiation regime. For quantitative details on all irradiation regimes, refer to the main text and Table 1. (**a**) Protons: Orange bars depict the proton bunches of the UPTD’s cyclotron at a fixed frequency of 106MHz with bunches of ns duration (bar width not to scale). Green bars represent the ns long laser-driven proton bunches at DRACO. The dark green bar illustrates single-bunch ZFE irradiation; the light green bars show accumulated dose delivery, featuring a realistic $20\text{s}$ time interval between bunches (not applied in ZFE study). (**b**) Electrons: The depicted ELBE electron bunches feature a bunch length of 5ps at a bunch frequency of 13MHz (bar width not to scale, on ns-scale bunches of different irradiation regimes are drawn next to each other for improved visibility). Five macro pulses of 63$\upmu$s length 40ms apart were applied in the $\text {ELBE}_\text {UHDR}^\text {syn}$ irradiation regime.

The accelerator infrastructure of the dresden platform comprises a variety of accelerator types. This section gives a short introduction to the accelerators used in the presented *in vivo* study with ZFEs, focusing on the temporal parameters of the applied dose. In total, seven different irradiation regimes were utilized in the experimental campaign (details below). Table 1 summarizes the accelerators’ diverse bunch parameters with respect to said irradiation regimes, showing that dose application over a wide range of peak and mean dose rates is feasible. The table additionally provides spatial and spectral beam properties such as beam diameter on the sample, energy spectrum, and linear energy transfer (LET). Fig. 2 visualizes the temporal sequences of delivered particle bunches for each respective accelerator and irradiation regime.

**Table 1:** Beam parameters of all ZFE irradiation experiments conducted across the dresden platform accelerator landscape.

		ELBE				UPTD		DRACO
Radiation type		Electrons				Protons		Protons
Kinetic energy	[MeV]	30				225		$<28$
LET	[keV/$\upmu$m]	0.274				0.417		$>3$
Beam diameter	[mm]	6.5				6.5		5
Bunch frequency	[MHz]	13				106		Single bunch
Bunch length at sample		5ps				2ns		20ns
Irradiation regime		${\textbf {ELBE}}_{\textbf {ref}}$	${{\textbf {ELBE}}}_{{\textbf {UHDR}}}^{{\textbf {iso}}}$	${{\textbf {ELBE}}}_{{\textbf {UHDR}}}^{{\textbf {syn}}}$	${{\textbf {ELBE}}}_{{\textbf {UHDR}}}^{{\textbf {max}}}$	${\textbf {UPTD}}_{{\textbf {ref}}}$	${\textbf {UPTD}}_{{\textbf {UHDR}}}$	${\textbf {DRACO}}_{{\textbf {single}}}$
Dose application time		240s	100ms	160ms	0.3ms	200s	115ms	20ns
Delivered bunches		$3.1 \cdot 10^9$	$1.3 \cdot 10^6$	4125	3935	$21.2 \cdot 10^9$	$12.2 \cdot 10^6$	1
Dose per bunch	[Gy]	$10.1 \cdot 10^{-9}$	$24.5 \cdot 10^{-6}$	$7.8 \cdot 10^{-3}$	$8.2 \cdot 10^{-3}$	$1.4 \cdot 10^{-9}$	$2.5 \cdot 10^{-6}$	$<30$
Mean dose rate	[Gy/s]	0.13	319	202	$1.1 \cdot 10^5$	0.15	262	$\sim 10^{9}$
Peak dose rate	[Gy/s]	$2.0 \cdot 10^3$	$4.9 \cdot 10^6$	$1.6\cdot 10^9$	$1.6 \cdot 10^9$	0.71	$1.2 \cdot 10^3$	$\sim 10^{9}$
Delivered macro pulses				5				
Macro pulse length				63$\upmu$s				
Macro pulse frequency	[Hz]			25				
Bunches per macro pulse				825				
${\dot{D}}_\text{macro}$	[Gy/s]			$1.0 \cdot 10^5$				

The presented data refers to the application of a radiation dose of $\sim 30\text{Gy}$ (exact dose values given in Table 2). A graphical representation of the accelerators’ temporal sequence of the beam bunches for the different irradiation regimes can be found in Fig. 2.

The University Proton Therapy Dresden (UPTD) at the University Hospital Carl Gustav Carus and OncoRay operates an isochronous cyclotron Cyclone 230 (Proteus Plus clinical PT facility, IBA) that provides temporally quasi-continuous proton beams with bunches of $2\text{ns}$ duration at a frequency of $106\text{MHz}$. In addition to the UPTD’s gantry room for patient treatment, the facility hosts and operates an experimental hall equipped with a horizontal fixed beamline and a scanning-nozzle beamline, which are both used for radiobiology and physics experiments^19,30,31^. There, proton beam parameters are adjustable beyond the clinical setting, enabling UHDR studies^32^. Strictly speaking, UPTD only represents the clinical branch of the facility, however, we here use UPTD to label the clinical cyclotron and experiments conducted with its proton beam at OncoRay’s fixed beamline, following the nomenclature of a previous publication^32^. The highest proton transmission from the cyclotron to the irradiation site is achieved for the maximum proton energy of $\sim 225\text{MeV}$, as used in the presented experiments. Irradiation was performed in the entrance region of the spectrally unmodulated proton beam, where mean dose rates of $\sim 300\text{Gy}/\text{s}$ were achieved at a beam current of $\sim 210\text{nA}$ at the irradiation site. For reference irradiation, protons of the same kinetic energy at a reduced beam current of $\sim 0.1\text{nA}$ yielded a mean dose rate of $0.15\text{Gy}/\text{s}$. Fig. 2a illustrates both irradiation regimes $(\text{UPTD}_\text{UHDR}, \text{UPTD}_\text{ref})$.

The laser-driven proton source DRACO^21^ (Dresden laser acceleration source) at the Helmholtz-Zentrum Dresden–Rossendorf uses ultra-short pulses of the petawatt laser DRACO to accelerate protons in a laser-generated plasma on the micrometer-scale. Laser-driven proton bunches feature a large energy-dependent divergence of $200-400\text{mrad}$ half opening angle and an exponentially decaying energy spectrum up to a maximum energy cut-off, here exceeding $70\text{MeV}$^33^. The proton beamline ALBUS-2S installed at DRACO shapes application-adapted homogeneous volumetric dose distributions of mm- to cm-scale at an in-air irradiation site from the spectrally-broad laser-driven proton bunches using the chromatic focusing of two pulsed solenoid magnets^34^. The proton source spectrum can be changed by adjusting the laser pulse energy. The magnetic field strength of the solenoids is also tunable. Combining the tunability of source spectrum and magnetic beam transport with passive beamline elements, such as apertures and scatter foils, enables to tailor the spectrum of the transported proton bunch and thus its depth dose distribution to match the specifics of the respective sample geometry. Proton bunch doses at the irradiation site can be adjusted from $300\text{mGy}$ to multi-$10\text{Gy}$ for a proton bunch length of $\sim 20\text{ns}$^35^, yielding peak dose rates exceeding $10^{9}\text{Gy}/\text{s}$. One aim of the ZFE study at DRACO was to simultaneously maximize the peak and the mean dose rate. Therefore, every ZFE sample was irradiated with a single proton bunch at the maximum available bunch dose ($\text{DRACO}_\text{single}$ in Fig. 2a). Fluctuations in the laser-plasma acceleration process result in a certain spread of the dose delivered to the ZFE samples as will be shown later. These bunch-to-bunch fluctuations can be mitigated by accumulative dose delivery to the sample by multiple bunches at the cost of the achievable mean dose rate. The repetitive generation of proton bunches from the laser-plasma acceleration source is ultimately limited by the repetition rate of the laser system, $1\text{Hz}$ in the case of DRACO. However, the operation of the ALBUS-2S beamline currently limits the repetition rate to one proton bunch per $20\text{s}$ ($\text{DRACO}_\text{accu}$ in Fig. 2a). Dose application to a sample by accumulation over several proton bunches hence yields mean dose rates of $\sim 1 - 60\text{Gy}/\text{min}$, depending on the bunch dose.

The research electron accelerator ELBE^36^ (Electron Linac for beams with high Brilliance and low Emittance) at the Helmholtz-Zentrum Dresden–Rossendorf provides electron beams with kinetic energies of $\le 40\text{MeV}$ with highly variable bunch parameters regarding their temporal sequence and dose per bunch^24,32,37^. Bunches of $5\text{ps}$ duration are delivered at a fixed frequency of $13\text{MHz}$ but can be modulated by grouping a selectable number of bunches into so-called macro pulses (see Fig. 2b). The time interval between these macro pulses can be tuned from $\sim \text{ms}$ to $\sim \text{min}$, modulating the bunch frequency by a superimposed macro pulse frequency. The dose per bunch is adjustable over six orders of magnitude. These capabilities allow ELBE to reproduce dose application characteristics of a multitude of accelerators, ranging from the conventional, quasi-continuous dose delivery over minutes of clinical machines to flexible UHDR delivery within micro- to milliseconds for FLASH research. This enables the direct comparison of reference and UHDR irradiation regimes at a single machine, eliminating variation between experiments conducted at different accelerators and at different times (e.g., consecutive experiments). For UHDR studies in ZFEs, an irradiation regime that maximizes the mean dose rate ($\sim 10^5\text{Gy}/\text{s}$) and the peak dose rate ($\sim 10^9\text{Gy}/\text{s}$) was implemented ($\text {ELBE}_\text {UHDR}^\text {max}$), marking the limits of the ELBE accelerator^24, 38^. The dose rates were gradually reduced following irradiation regimes that mimic UHDR dose delivery at a clinical isochronous proton cyclotron (quasi-continuous over $\sim 100\text{ms}$, $\text {ELBE}_\text {UHDR}^\text {iso}$) and a clinical proton synchrocyclotron ($\sim {\upmu \text {s}}$ macro pulses with $\sim \text{ms}$ repetition time, $\text {ELBE}_\text {UHDR}^\text {syn}$)^32^. These regimes were delivered at comparably high mean dose rates of few $100\text{Gy}/\text{s}$ but differ with respect to the temporal beam structure and consequentially peak dose rate with $10^{6}\text{Gy}/\text{s}$ for $\text {ELBE}_\text {UHDR}^\text {iso}$ and $10^{9}\text{Gy}/\text{s}$ for $\text {ELBE}_\text {UHDR}^\text {syn}$.

**Figure 3:**
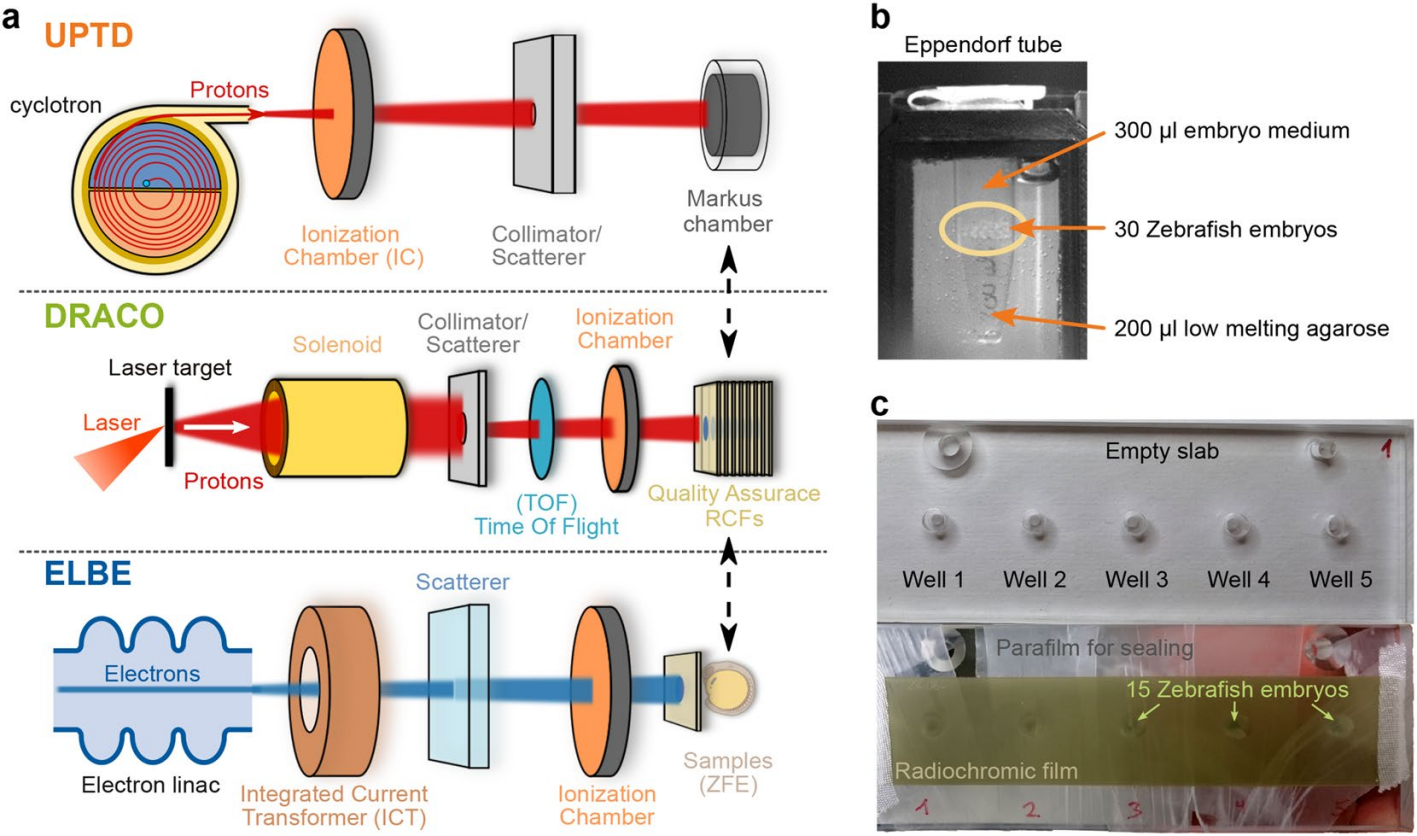
(**a**) Simplified sketches of the irradiation setups at the dresden platform accelerators UPTD (fixed beamline), DRACO, and ELBE. The dashed arrows indicate the interchangeability of samples or detectors at the end of each beamline. (**b**) Photograph of a ZFE-filled Eppendorf tube used for irradiation studies at UPTD and ELBE. (**c**) Picture of the slabs used for ZFE irradiation at DRACO. The top shows five empty wells, the bottom ZFE-filled wells sealed with Parafilm and radiochromic film in front.

### Biological model

ZFEs as a small animal vertebrate model are increasingly applied as a model for acute normal tissue reactions for UHDR investigations^24,28^ due to their high number of offspring and their small size ($1\hbox {mm}$ diameter 24 h post fertilization (hpf)). The latter enables meaningful radiobiological studies at limited beam spot sizes^18,27^. Morphometric alterations such as the embryo length serve as radiobiological endpoints that are easily detected by light microscopy^27^.

As part of the dresden platform, a standardized animal experiment protocol was designed according to the European Parliament and Council (EU Directive 2010/63/EU) on the protection of animals used for scientific purposes and in accordance with German legislation on the care and use of laboratory animals. This protocol ensures similar workflows and experimental conditions across the irradiation setups of the dresden platform (Fig. 3a) and ensures comparability of the radiobiological results. A brief description of the protocol is given below, more detailed information on general embryo handling and staging can be found elsewhere^18,39,40^.

Wildtype ZFEs (strain AB) and E3 embryo medium^39^ were sourced from the Center for Regenerative Therapies of TUD Dresden University of Technology. The embryos were transported to the respective facilities under careful temperature maintenance and kept at room temperature ($23 - 25{^\circ \hbox {C}}$) until and during all irradiations. The experiments were performed in the pharyngula stage of the embryos, i.e., starting at 24 hpf and finishing within 6 hours. To minimize the influence of embryonic development on radiosensitivity during this time, UHDR and reference irradiations were performed in alternating order if possible.

Because of an earlier UHDR study showing that the protecting effect of ultra-high dose rates is reduced if the partial oxygen pressure ($\text{pO}_{2}$) in the samples is too high during irradiation^24^, all studies were performed under controlled low $\text{pO}_{2}$ of less than $10\text{mmHg}$. At ELBE and UPTD, this level was reached by sealing about 30 embryos in a $0.5\hbox {ml}$ Eppendorf tube filled with $200{\upmu \hbox {l}}$ low melting agarose as a base layer to have all ZFE samples at the same vertical position and $\sim 300{\upmu \hbox {l}}$ E3 embryo medium, approximately 1*h* before irradiation^24^ (Fig. 3b). The actual $\text{pO}_{2}$ in the medium around the embryos was controlled in parallel in control samples using the OxyLite sensor (Oxford Optronix Ltd).

For irradiations at DRACO, customized acrylic (PMMA) slabs (Fig. 3c) were manufactured in-house and used instead of Eppendorf tubes (Fig. 3c). The DRACO irradiation setup employed what we call a “multi-well slab”. This slab contained five milled cylindrical holes (or wells) of $4.5\text{mm}$ diameter and $3.5\text{mm}$ depth for consecutive irradiation. Comparative measurements to investigate the influence of the sample holder were conducted at UPTD. Here, a single-well slab was employed that otherwise exhibited the same geometry and material composition. 15 ZFEs were placed in each hole of the PMMA slab. The holes were then filled up with E3 and sealed with tightly wrapped Parafilm. Due to the smaller volume, the necessary sealing time to achieve low $\text{pO}_{2}$ before irradiation had to be shortened. Exemplary, sealing times of approximately $15 - 30\text{min}$ before irradiation were applied for the slab setting at UPTD and DRACO.

After irradiation, the ZFEs were separated in 96-well plates and maintained under standard conditions ($28{^\circ \hbox {C}}$) for up to four days including medium exchange every other day. On the fourth day post-irradiation, pictures were taken from each surviving embryo before termination and fixation in $2{\%}$ paraformaldehyde (Sigma-Aldrich, Burlington, MA, USA) for further analysis using a microscope (Axiovert S100, 25× magnification, Zeiss). From these pictures, individual embryo lengths were measured by applying ZEN, Version 2.6 (Zeiss).

### Dose application and dosimetry

Comparable irradiation conditions for the ZFEs across the accelerator infrastructure of the dresden platform, irrespective of the irradiation regime, require infrastructure-specific solutions for beam transport and dose application whereas common beam monitoring and dosimetric concepts can be applied. Figure 3a illustrates the respective setups at UPTD, DRACO, and ELBE.

To maximize the achievable dose rates, beam transport setups at all accelerator facilities were optimized for highest transmission and beam spots at the irradiation sites were kept small. Accordingly, only thin scatterers were applied to homogenize the radiation fields ($15\text{mm}$ of PMMA at UPTD, $100{\upmu \hbox {m}}$ lead at DRACO, $2\hbox {mm}$ of PMMA at ELBE). At ELBE and UPTD, irradiation setups were tailored to deliver homogeneous dose distributions ($<10{\%}$ lateral and depth dose variation) of $3\text{mm}~\mathrm {(H)} \times 6.5\text{mm}~\mathrm {(W)} \times 6.5\text{mm}~\mathrm {(D)}$ size, matching the geometry of $0.5\text{ml}$ Eppendorf tubes. Irradiations were performed at $\sim 30\text{Gy}$. To achieve a similar dose at DRACO in a single bunch, the irradiated volume was reduced, which made the adaptation of the irradiation setup to the PMMA slab setup necessary. The dose was applied homogeneously to a cylindrical volume of $5\text{mm}$ diameter and $4\text{mm}$ depth^26^.

The volumetric dose distributions were controlled on a daily basis. At UPTD, the Lynx scintillation detector and the Giraffe detector (both IBA Dosimetry GmbH) were applied for the lateral and depth dose distributions, respectively. At DRACO, stacks of radiochromic films (RCF, GafChromic EBT3) were irradiated^26^. At ELBE, the lateral beam profile was measured via a phosphorescent screen imaged by a digital camera. The dose buildup of the $30\text{MeV}$ ELBE electron beam over the sample depth was characterized with RCF stacks^24^.

The total dose delivered to each ZFE sample was derived from the retrospective readout of a single RCF positioned in front of each sample during irradiation. The RCF also provided the lateral dose homogeneity. Calibration of the RCF for electron and proton radiation in $10 \times 10{\hbox {cm}^2}$ homogeneous fields was performed at a clinical linac and the UPTD cyclotron, respectively.

In addition to retrospective dose evaluation, online monitoring of dose delivery was realized at each accelerator site by means of transmission ionization chambers (IC, all readout with UNIDOS electrometers, PTW) in combination with a second online detector system. At UPTD, a Bragg peak IC (T34070-2,5, PTW) positioned close to the beamline provided an online ionization charge measurement and was cross-calibrated daily against the advanced Markus IC (34045, PTW) placed at the sample position. Here, recombination losses of about $2{\%}$ were estimated and taken into account for the UHDR irradiation regime ($\text{UPTD}_\text{UHDR}$)^41^. Moreover, the beam monitor chamber (34058, originally an OEM product, PTW) integrated into the proton beam exit served as additional control and was therefore included in the cross-calibration procedure.

At DRACO, a transmission IC (7862, PTW) was applied for relative charge measurements only, as the UHDR led to strong recombination losses of the IC and prevented a meaningful cross-calibration against, e.g., a Markus IC (34045, PTW) at the sample position. Additionally, an online transmission time-of-flight (ToF) spectrometer monitored the proton bunch spectrum, length, and intensity for each dose delivery to a ZFE sample^35^. Based on the measured bunch spectra in combination with Monte Carlo simulations of the beam transport from the ToF spectrometer to the sample position, the depth dose distributions for each sample irradiation were reconstructed and hence also monitored^35^.

At ELBE, the transmission IC (7862, PTW) was operated in combination with an integrated current transformer (ICT: ICT-CF 4.5”/34.9-070-05:1-UHV, Bergoz Instrumentation; readout: Oscilloscope DPO 7254; Tektronix) at the beam exit. The ICT controlled bunch charge and sequence constancy for UHDR beam delivery. The charge readout of the transmission IC was cross-calibrated against a Markus IC (34045, PTW) placed at the sample position. Cross-calibration was performed in the reference irradiation regime $\text{ELBE}_\text{ref}$ to circumvent recombination effects. Combining the measured dose (dose uncertainty $<10{\%}$) and the temporal beam structure, final values for mean and peak dose rates were derived (see Table 1).

### Oxygen depletion measurements

In addition to controlling the initial partial oxygen pressure $\text{pO}_{2,\hbox {start}}$ in the ZFE samples prior to irradiation, as described above, the online measurement of oxygen depletion during irradiation is a vital feature of radiobiological studies in the UHDR regime. The TROXSP5 sensor (PyroScience GmbH) can measure oxygen dissolved in liquids non-invasively via stimulated fluorescence. Such oxygen depletion measurements were established at the dresden platform using water phantoms as previously described in^29,32,38^. Here we extended this method to in-sample measurements for proton irradiations in the PMMA slab setup at UPTD and DRACO.

The sensors were glued into the slab wells using silicone before the wells were filled with ZFEs and sealed with Parafilm. Holes on the rear side of the slab held optical fibers connected to the FireSting-O2 oxygen meter (FSO2-C4, PyroScience GmbH). This method has a time resolution of $\sim 400\hbox {ms}$. The sensors were calibrated in water at 0% and 21% oxygen concentration, with temperature and pressure corrections in accordance with the manufacturer’s guidelines.

## Results and discussion

**Figure 4:**
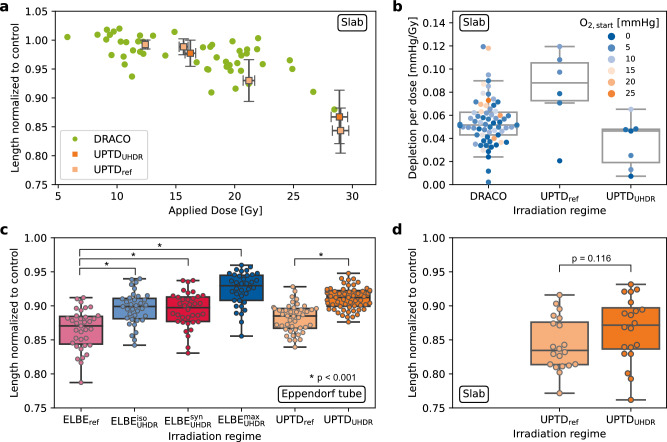
Summary of all ZFE experiments conducted across the dresden platform. The used sample holders are stated for every sub-figure. ZFE length data were normalized to respective unirradiated controls to correct for batch-dependent size differences and are presented (if applicable) in the form of box plots for comparison. The box ranges from the lower to the upper quartile of the data set. The horizontal line marks the median. The height of the box is the interquartile range (IQR). Whiskers mark the minimum and maximum values within the data set excluding outliers (distance $>1.5\times \text{IQR}$ from the lower/upper quartile). (**a**) Results of ZFE proton irradiation studies. Green dots represent individual single-bunch irradiations at DRACO with delivered doses between $\sim 6\text{Gy}$ and $\sim 28\text{Gy}$. Orange squares show grouped data taken at UPTD with $1\sigma$ standard deviation. Prescribed dose values have been varied to allow for a comparison to the DRACO dose-response curve. (**b**) Corresponding oxygen depletion per dose at the proton accelerators as a function of the measured initial oxygen level $\text{pO}_{2,\hbox {start}}$. (**c**) and (**d**) Results of ZFE irradiation studies conducted at UPTD and ELBE at $\sim 30\text{Gy}$ delivered dose (details in Table 2), data in (**d**) corresponds to the rightmost data points shown in (**a**). Comparing the identically scaled UPTD Eppendorf tube data and the slab data reveal a larger variation in radiation damage for the slabs.

**Table 2:** Compilation of experimental results obtained at ELBE and UPTD^32^.

	Sample holder	Irradiation regime	Samples [n]	Dose [Gy]	Embryo length [$\upmu$m]
**ELBE**	Eppendorf	${\textbf {ELBE}}_{{\textbf {ref}}}$	35	31.5 ± 0.6	3403 ± 121
		${{\textbf {ELBE}}}_{{\textbf {UHDR}}}^{{\textbf {iso}}}$	35	31.9 ± 0.5	3526 ± 99
		${{\textbf {ELBE}}}_{{\textbf {UHDR}}}^{{\textbf {syn}}}$	36	32.3 ± 0.6	3517 ± 110
		${{\textbf {ELBE}}}_{{\textbf {UHDR}}}^{{\textbf {max}}}$	36	32.1 ± 0.6	3637± 107
		**Controls**	38		3932 ± 41
**UPTD**	Eppendorf	${\textbf {UPTD}}_{{\textbf {ref}}}$	44	30.2 ± 0.6	3505 ± 105
		${\textbf {UPTD}}_{{\textbf {UHDR}}}$	56	30.1 ± 0.8	3624 ± 85
		**Controls**	22		3974 ± 81
	Slab	${\textbf {UPTD}}_{{\textbf {ref}}}$	20	29.0 ± 0.7	3400 ± 157
		${\textbf {UPTD}}_{{\textbf {UHDR}}}$	20	28.9 ± 0.7	3488 ± 189
		**Controls**	11		4030 ± 29

UPTD results are broken down by sample holder. The embryo body length is given as mean value ± standard deviation (sd) over all samples [n].

The ZFE irradiation experiment at the DRACO laser-driven proton source with synchronous in-sample oxygen depletion analysis completes a set of previously conducted ZFE studies at ELBE and UPTD (Table 1)^32^. The irradiation with DRACO protons was performed in the $\text {DRACO}_\text {single}$ irradiation regime, delivering single bunches in the dose range of $6 - 28\text{Gy}$ at identical peak and mean dose rates of $\sim 10^9\text{Gy}/\text{s}$ (Fig. 4a/b).

Previous results for ELBE electrons and UPTD protons for single-fraction ZFE irradiations in the Eppendorf tube setting with doses of $\sim 30\text{Gy}$ are reproduced from^32^ in Fig. 4c (see Table 2 for details). Applying the irradiation regimes detailed in Table 1, a clear beneficial effect of UHDR electron and proton treatment is demonstrated, as expressed by longer embryo lengths compared to the respective reference irradiation. All embryo lengths presented in Fig. 4 were normalized to the respective controls to rule out batch differences in embryo body length, in particular when comparing different campaigns. The data for electron irradiation show that an increase of the mean dose rate from a few $100\text{Gy}/\text{s}$ ($\text {ELBE}_\text {UHDR}^\text {iso}$, $\text {ELBE}_\text {UHDR}^\text {syn}$) to $10^5\text{Gy}/\text{s}$ ($\text {ELBE}_\text {UHDR}^\text {max}$) results in even longer embryos, i.e., better protection against radiation damage. The peak dose rate, on the other hand, does not influence the embryo lengths, as verified by equal embryo lengths for the $\text {ELBE}_\text {UHDR}^\text {iso}$ and $\text {ELBE}_\text {UHDR}^\text {syn}$ irradiation regime. Previous complementary measurements of the oxygen depletion in a water phantom for the irradiation regimes under consideration yielded an anti-correlation of oxygen depletion with dose rate^29,38^. In detail, Jansen et al.^38^ found that the oxygen depletion decrease with increasing mean and peak dose rates. This finding contradicts the theory that transient hypoxia as a result of increased oxygen depletion during UHDR irradiation^7,11,42^ is the driving mechanism behind FLASH.

Irradiations at DRACO required two methodical adjustments compared to studies at ELBE and UPTD. Firstly, as discussed above, otherwise applied Eppendorf tubes were replaced by slabs with the capability for synchronous in-sample oxygen measurements during each irradiation. Secondly, to manage bunch-to-bunch intensity fluctuations inherent to laser-driven sources, a dose-response curve for the embryo length was measured instead of the ZFE response to a single dose value (Fig. 4a). The dose-response curve indicates a beneficial effect of proton irradiation at $10^9\text{Gy}/\text{s}$ peak and mean dose rate compared to the minute-long reference irradiation at UPTD ($\text {UPTD}_\text {ref}$) for doses $\gtrsim 20\text{Gy}$. The results are a first hint that a protective effect of UHDR proton irradiation is also present for dose application in a single bunch at an escalated dose rate of $\sim 10^{9}\text{Gy}/\text{s}$, as observed for lower UHDRs. For comparison, ZFE proton treatment in the $\text {UPTD}_\text {UHDR}$ regime (here at $360\text{Gy}/\text{s}$ mean dose rate) was added (dark orange dots in Fig. 4a).

However, there are systematic limitations in the conducted irradiation study that prevent conclusive radiobiological results at this stage. For one, there are differences in radiation quality regarding the LET between the experiments at DRACO (spread-out Bragg peak, $\text{LET}_\text{DRACO}$ > 3keV/$\upmu$m) and UPTD (entrance channel, $\text{LET}_\text{UPTD}$ $=$ 0.417keV/$\upmu$m). Furthermore, at DRACO a maximum dose of $28\text{Gy}$ was delivered but the majority of dose values are below $20\text{Gy}$, despite accelerator operation at the highest achievable performance level^35^ at the time of the experiment. More importantly, the slabs applied as sample holders were identified to cause fluctuations in the oxygen level prior to irradiation. The effect is directly visible in the in-sample measurements of the initial oxygen level and oxygen depletion taken with multi- and single-well slabs at DRACO and UPTD (see Fig. 4b). Particularly for DRACO, both the $\text{pO}_{2,\hbox {start}}$ values measured shortly before ZFE irradiation and the oxygen depletion per dose vary strongly. $\text{pO}_{2,\hbox {start}}$ values of up to $25\text{mmHg}$ were observed, clearly exceeding the target value of $10\text{mmHg}$ at time of irradiation. However, the anti-correlation between dose rate and oxygen depletion, as measured before^29,42^, is confirmed.

This finding raises the question of whether the slab sample holder affects the radiobiological endpoint in direct comparison to the Eppendorf tube setup. Hence, ZFEs were irradiated in single-well slabs (Fig. 4d) and Eppendorf tubes (Fig. 4c) in parallel at UPTD in alternating order to avoid effects of embryo aging over time. As Fig. 4c/d and Table 2 show, the ZFEs treated in slabs are significantly shorter (p$=0.011{}$ for $\text {UPTD}_\text {ref}$, p$=0.005{}$ for $\text {UPTD}_\text {UHDR}$). The ZFEs furthermore feature a higher variability in body length, as expressed by a high standard deviation (see Table 2). The beneficial effect of UHDR proton irradiation, which was significantly observed in the Eppendorf setting (p$<0.001{}$) could not be verified for the slabs. However, the general trend of less damage for increased mean dose rate, i.e., longer embryos, was observed (p$=0.116{}$). For all proton irradiations, the endpoints were compared between reference and UHDR by the two-sided t-test for independent samples.

It is known, e.g., from xenograft studies in mice^43^, that variable oxygen levels during irradiation result in a different radiobiological outcome. We identified the imperfect sealing of the slabs as the cause of non-reproducible oxygen conditions in the sample and thereby as the dominant source for the strong fluctuations in embryo body length. On the one hand, the lid attached to an Eppendorf tube allows for tight and reproducible sealing of the sample volume resulting in a continuous reduction of $\text{pO}_{2}$ in the embryo medium by embryonic oxygen consumption^24^. On the other hand, the wells of the slabs were sealed by wrapping Parafilm around the slab, which might have induced small air bubbles or gaps, as the Parafilm’s stretching and adhesion could not be standardized. Moreover, reusing the slabs during the experiment may have left a thin wet film on the plastic, further reducing the adhesion of the Parafilm. These assumptions match the observed variations in the $\text{pO}_{2,\hbox {start}}$ level and oxygen depletion for the multi- and single-well slabs as employed at DRACO and UPTD (see Fig. 4b), with the larger multi-well slab being more prone to fluctuation. Besides the sealing effect, it needs to be taken into account that the presented measurements were performed in-sample, i.e., in a biological system instead of a water phantom, resulting in an inherently higher $\text{pO}_{2}$ variance^38^. This may also contribute to the high fluctuations in $\text{pO}_{2}$, as it was also observed in other *in vivo* systems^42,43^. A direct effect of the different $\text{pO}_{2}$ levels could be excluded referring to a previous work^24^, where the body length of the treatment controls (sham irradiation) of the low (below 10 mmHg) and high $\text{pO}_{2}$ groups are comparable to each other and to the embryo lengths of the laboratory control that remain under atmospheric $\text{pO}_{2}$ levels. Likewise, in the present work, the embryo length of the controls maintained in slabs is comparable to that observed in Eppendorf tubes (Table 2) and to the laboratory control (mean body length of the embryos was (3966 ± 68)$\upmu$m). Besides $\text{pO}_{2}$, the environmental temperature and bystander signaling are two factors that potentially influence the response of the ZFE to radiation. Studying the temperature-dependent response to UV-B radiation, Aksakal and Ciltas^44^ demonstrated that ZFE irradiated at $24{^\circ \hbox {C}}$ show more damage and delayed development compared to those irradiated at standard conditions of $28{^\circ \hbox {C}}$. In their study, the ZFEs were permanently maintained at the lower temperature, which is known to slow down development considerably^40^. In the experiments presented here, however, the ZFEs were kept at room temperature only for a limited time and otherwise under standard conditions, reducing the temperature influence on ZFE development after irradiation. In addition, residual environmental influences affect all samples to the same extent, since laboratory controls, sham irradiated, and irradiated samples of conventional and ultra-high dose rates were treated identically. Bystander signaling, i.e., a response of unirradiated ZFEs after pairing them with irradiated ZFEs or their medium, is a well-described phenomenon for zebrafish embryos (e.g.^45^). In order to avoid bystander signaling in the present work, the dose homogeneity of the irradiation fields was carefully controlled to ensure the same treatment doses for all ZFEs of one irradiation sample (up to 30 ZFEs). Post irradiation, the ZFE samples were separated and stored in unirradiated medium in 96-well plates, thus preventing bystander signaling. Moreover, the medium exchange in the Eppendorf tubes and slabs after irradiation inhibits the signaling between subsequent ZFE samples, minimizing the chance of bystander effects.

Assessing the presented results from a technological point of view, the data underline the usability of the DRACO system for radiobiological studies at UHDR in a FLASH-relevant setting. With this capability, the available dose rate range for protons within the dresden platform is extended to $\sim 10^{9}\text{Gy}/\text{s}$. In terms of radiobiological outcome, the observed protective effect for ZFEs for irradiation with escalated dose rates of $\sim 10^{9}\text{Gy}/\text{s}$ is an indication against a possible saturation of the FLASH effect up to the applied mean dose rates. The comparable depletion observed in the $\text {DRACO}_\text {single}$ and $\text {UPTD}_\text {UHDR}$ regimes hints towards saturation of the radiolytic oxygen depletion at escalated dose rates. Both findings show the need for further radiobiological studies over a broad range of dose rates^1^ to reveal the underlying dependencies^10^ and to generate experimental reference data for radiochemical simulations to support experimental observations^8,46^.

For DRACO, follow-up experiments providing a conclusive radiobiological outcome require higher dose values with increased reproducibility, calling for concentrated efforts toward further maturation of the laser-driven proton accelerator technology. Additionally, it is favorable to have comparable LET values across proton irradiations. To achieve this, UPTD has recently established a spread-out Bragg peak irradiation scheme capable of delivering clinical dose rates and UHDR^20^. However, the most important improvement concerns the multi-well slab setting. It can be assumed that the strongly varying oxygen concentrations in the corresponding ZFE samples correlate with the observation of increased variability in body lengths, as directly observed for the single-well slab setting at UPTD. Improved and reproducible slab sealing can be achieved with biocompatible adhesive foils, as applied for plate sealing in qPCR, or plastic caps for the slab holes. The reliability of such approaches will be tested independently from irradiation studies via measurements of the oxygen concentrations.

## Conclusion

**Figure 5:**
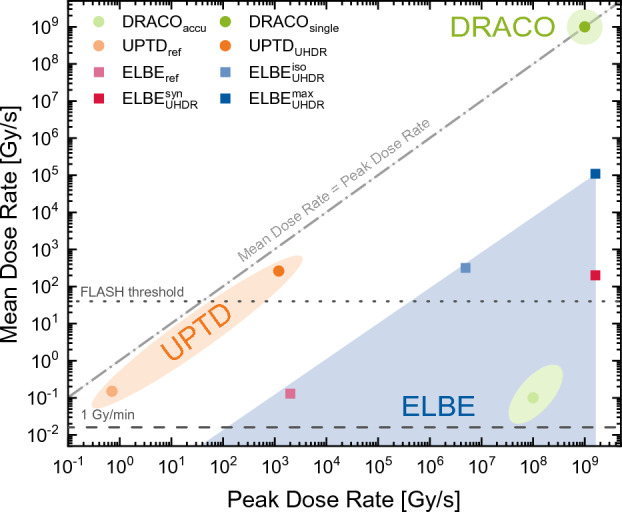
Accessible peak and mean dose rate combinations of the dresden platform accelerator portfolio. Individual data points (dots for protons and squares for electrons) correspond to the presented irradiation regimes and the data given in Table 1. The shaded areas represent other possible peak and mean dose rate combinations made available at the accelerators.

In conclusion, the presented results of the concerted radiobiological *in vivo* studies applying the zebrafish embryo model qualify the dresden platform as a research hub for systematic investigations on UHDR radiation in the context of the FLASH effect. As shown, this firstly concerns the provision of a broad range of dose application parameters for peak and mean dose rates (Fig. 5) but extends to solutions for dose delivery, dosimetry, and beam monitoring and includes the availability of a biological research infrastructure and suitable biological models.

The dresden platform highlights that translating the benefits of UHDR irradiation to FLASH RT for an improved quality of life of patients is a task heavily dependent on research accelerator infrastructure and novel acceleration schemes. As illustrated in Fig. 5, dose application parameters beyond the predicted FLASH threshold are achieved at the experimental arm of UPTD, the ELBE accelerator, and the DRACO laser-driven proton source. Here, particularly the example of DRACO shows that even accelerators in the early development phase can be prepared for sophisticated radiobiological *in vivo* studies and then open the door to a previously inaccessible but highly FLASH-relevant parameter range. On the other hand, qualification of novel accelerators for FLASH research is most efficient when embedded in an existing research infrastructure including established reference accelerators, as given at the dresden platform.

With the achieved performance level, the next step is the investigation of more complex biological models, e.g., rodents. At DRACO, the first successful pilot study based on a mouse ear tumor model has been performed recently^26^. At OncoRay, radiobiological studies in mice for translational research are well established, from biological models^27^ to irradiation and imaging setups^47,48^. As a result, the knowledge on UHDR effects gained at research hubs such as the dresden platform will hopefully help to identify clinically relevant and implementable FLASH dose application parameters and contribute to the design of future clinical FLASH RT machines.

## Data Availability

The source data for the ZFE slab irradiations at Draco and UPTD, including the measured embryo lengths, applied doses, and observed pO2 starting values are available via RODARE at https://doi.org/10.14278/rodare.2381. The ZFE data obtained in the Eppendorf tube setting (ELBE, UPTD) are taken from Karsch et al.^32^. All other data that support the plots within this paper and other findings of this study are available from the corresponding author upon reasonable request.
